# Inhibition of the Host Proteasome Facilitates Papaya Ringspot Virus Accumulation and Proteosomal Catalytic Activity Is Modulated by Viral Factor HcPro

**DOI:** 10.1371/journal.pone.0052546

**Published:** 2012-12-27

**Authors:** Nandita Sahana, Harpreet Kaur, Basavaraj, Fatima Tena, Rakesh Kumar Jain, Peter Palukaitis, Tomas Canto, Shelly Praveen

**Affiliations:** 1 Division of Biochemistry, Indian Agricultural Research Institute, New Delhi, India; 2 Division of Plant Pathology, Indian Agricultural Research Institute, New Delhi, India; 3 Department of Horticultural Science, Seoul Women’s University, Seoul, South Korea; 4 Centro de Investigaciones Biológicas, CIB, CSIC, Madrid, Spain; Friedrich-Alexander-University Erlangen-Nurenberg, Germany

## Abstract

The ubiquitin/26S proteasome system plays an essential role not only in maintaining protein turnover, but also in regulating many other plant responses, including plant–pathogen interactions. Previous studies highlighted different roles of the 20S proteasome in plant defense during virus infection, either indirectly through viral suppressor-mediated degradation of Argonaute proteins, affecting the RNA interference pathway, or directly through modulation of the proteolytic and RNase activity of the 20S proteasome, a component of the 20S proteasome, by viral proteins, affecting the levels of viral proteins and RNAs. Here we show that MG132, a cell permeable proteasomal inhibitor, caused an increase in papaya ringspot virus (PRSV) accumulation in its natural host papaya (*Carica papaya*). We also show that the PRSV HcPro interacts with the papaya homologue of the Arabidopsis PAA (α1 subunit of the 20S proteasome), but not with the papaya homologue of Arabidopsis PAE (α5 subunit of the 20S proteasome), associated with the RNase activity, although the two 20S proteasome subunits interacted with each other. Mutated forms of PRSV HcPro showed that the conserved KITC54 motif in the N-terminal domain of HcPro was necessary for its binding to PAA. Co-agroinfiltration assays demonstrated that HcPro expression mimicked the action of MG132, and facilitated the accumulation of bothtotal ubiquitinated proteins and viral/non-viral exogenous RNA in *Nicotiana benthamiana* leaves. These effects were not observed by using an HcPro mutant (KITS54), which impaired the HcPro – PAA interaction. Thus, the PRSV HcPro interacts with a proteasomal subunit, inhibiting the action of the 20S proteasome, suggesting that HcPro might be crucial for modulating its catalytic activities in support of virus accumulation.

## Introduction

Plant viruses invade their host cells and utilize the cellular pathways of the host to support various aspects of their infection cycle. To achieve this goal, viruses must neutralize the multifaceted defense mechanisms of the host to make them susceptible to viral propagation and movement. Several studies have shown the involvement of the protein degradation machinery, the ubiquitin/26S proteasome system (UPS), in plant–virus interactions during infection. The protein degradation machinery of eukaryotes is comprised of a barrel-shaped, 20S core element (20S proteasome). It is primarily composed of four stacked rings, consisting of seven α subunits forming each of the two outer rings, comprising the central chamber, and seven β subunits forming each of the two inner rings [1], [2] together comprising one percent of the total cellular proteins [3]. In eukaryotes, the two inner β rings are the core of proteolytic activities associated with chymotrypsin-like, trypsin-like and caspase-like activities, according to their specificity. These core elements, when associated with either one or two 19S regulatory particles define a 26S proteasome complex. The complex is involved in an ubiquitin- conjugation and protein degradation pathway in an ubiquitin-dependent or -independent manner involving an ATP-dependent cascade of three enzymes [4]–[6]. Two different activities of the 20S proteasome can play major roles in the molecular interactions between host and virus, the protease activity [7] and the associated RNase activity [8], [9]. The proteolytic and RNase activities of the 20S proteasome also affect the levels of viral proteins and RNAs. The vulnerability of viral proteins to proteolytic degradation depends upon their stability, which is based on the presence of PEST sequences as shown in case of the RNA-dependent RNA polymerase (RdRp) protein (66 K) of TYMV [10]. The viral proteins involved (mostly silencing suppressors) can interact with host RNA silencing-effector Argonaute proteins and facilitate the degradation of the latter through the 20S proteasome, thus affecting the RNA silencing defense response [11]. Alternatively, viral proteins can also interact directly with the 20S proteasome components, modulating their catalytic activities [12]. As shown by different groups, P25 of potato virus X (PVX) and P0 of poleroviruses were reported to degrade Argonaute proteins, affecting the RNA silencing machinery [11], [13], whereas the helper component protease (HcPro) of lettuce mosaic virus (LMV) and potato virus Y (PVY) were found to interact directly with different subunits of the 20S proteasome [14], [15]. Jin and his coworkers [14] found that the HcPro of PVY could interact with the PAA (α1), PBB (β2) and PBE (β5) subunits of the *Arabidopsis thaliana* 20S proteasome, but not with the PAE (α5) subunit, containing the RNase activity, while Dielen and coworkers [15] found that the LMV HcPro could interact with the *A. thaliana* PAE subunit.

Potyviral HcPro is a multifunctional protein, essential to the infection process [16]–[18]. Although HcPro has several other important functions in the viral infection cycle, such as aphid transmission [19], genome amplification, cell-to-cell and long-distance movement [20] and suppression of the RNA silencing defense responses [21], little is known about the links between the different activities of this protein. However, different functions of the potyviral HcPro have been mapped to different regions of this protein. For example, the protease function was mapped to the C-terminus [18], which is independent of the RNA silencing suppressor function [22] and the aphid-transmission function [23]–[24], and the sequences interacting with the three subunits of the 20S proteasome have been mapped to the N-terminal region [14].

Identification of selective proteasome inhibitors has allowed cell biologists to define the importance of the ubiquitination machinery in plant responses to the majority of biotic stresses, especially in plant-microbe interactions [25]. One of these inhibitors, MG132, a peptide aldehyde, inhibits the 20S proteasome chymotrypsin-like activity in a strong, reversible and cell-permeable manner. Although various groups have demonstrated the importance of the 20S proteasome in altering the levels of plant viral RNAs as well as proteins [9], [26], very similar to animal viruses [8], [27]–[29], there has been no correlation established between proteasome inhibition and virus accumulation patterns in infected plants.

Here, we have examined the effects of MG132 on the accumulation of papaya ringspot virus (PRSV) in papaya (*Carica papaya*). In addition, using the yeast two-hybrid and bimolecular fluorescence complementation (BiFC) assays, we determined whether PRSV HcPro could interact with the papaya homologues of *A. thaliana* proteasome subunits PAA and PAE, using both wild-type (wt) HcPro and two mutants, to identify specific domain required for the interactions. Finally, we also analyzed the catalytic activities of the 20S proteasome in the presence of HcPro.

## Materials and Methods

### Mechanical Inoculation of PRSV

*C. papaya* plantlets were grown under glasshouse conditions at 25–28°C for one month prior to sap inoculation with PRSV (strain P). One gram of leaf tissues from PRSV-infected plants was ground in 0.1 M potassium phosphate buffer, pH 7.0. Plants were dusted with Carborundum and PRSV-infected leaf extract was inoculated on the youngest fully-expanded leaves of each plant as described by Mangrauthia and coworkers [30].

### Detection of PRSV in Inoculated Plants

PRSV infection was detected and quantified serologically using the plate trapped-antigen enzyme-linked immunosorbant assays (ELISA) method [30] in Maxisorb microtiter plates (Nunc, Roskilde, Denmark), using 0.1 g fresh leaf tissue. All incubations were performed at room temperature, unless stated otherwise. The wells were washed three times between each step with phosphate buffered saline (PBS) plus 0.05% Tween 20 (PBST). Leaf samples were prepared in coating buffer (0.05 M sodium carbonate, pH 9.6, 1∶10 w/v) and were incubated overnight at 4°C. Bound virus particles were detected using PRSV antiserum (produced in-house), with a dilution of 1∶1000 in PBS. Goat anti-rabbit alkaline phosphatase conjugated secondary antibody (Sigma, St Louis) was used at a dilution 1∶30,000. The interaction was detected using *p*-nitrophenyl phosphate (1 mg/mL) substrate (Sigma) and was quantified with a Dynatech MR 7000 plate reader at an absorbance of 410 nm. Samples were considered positive when the absorbance exceeded twice the mean of the absorbance values for the appropriate healthy controls.

### Proteasome Inhibition Using MG132

MG132 (50 µM in 0.02% DMSO) was applied to the two young emerging leaves of a total of 15, one month-old papaya plants for each treatment [11], two hours prior to mechanical inoculation with extracts from papaya plant tissues infected with PRSV. Leaf samples from seven plants were then collected at various days post inoculation (dpi), both from MG132-treated and DMSO-treated control plants, for ELISA and quantitative reverse-transcription polymerase chain reaction assays (qRT-PCR), to determine the virus titer and levels of viral RNA, respectively. Viral titer was estimated by DAC-ELISA and viral RNA by qRT-PCR using 3′PRSV F/3′PRSV R primer set. To determine the effects of inhibition of the 20S proteasome on the accumulation of total ubiquitinated plant proteins, fully expanded papaya leaves were treated with the same concentration of MG132 before samples were collected at 24 hours post treatment. The remaining plants were used for photographs and to score the symptoms. Each experiment was repeated thrice.

### qRT-PCR Analysis

Samples of papaya leaves were collected at 1, 2, 4, 7, 9, 11 and 15 dpi with PRSV (both from MG132-treated and DMSO-treated control plants). Total RNA was isolated from 0.1 g of leaf tissue using an RNase Easy Plant Minikit (Qiagen) and was reverse transcribed to cDNA using random hexamer primers (Fermentas cDNA synthesis kit), as per the manufacturer’s protocol. The cDNA was then subjected to conventional PCR and qPCR. Amplification was done using 3′PRSV F and 3′PRSV R primers, based on sequences of the 3′ region of the PRSV genome, within the CP-coding region. The primers for qPCR were validated using gel electrophoresis of PCR amplicons and by the presence of single peaks in the melting curve. The qPCR was done using 25 ng of cDNA, 40 nM forward and reverse primer each and 1X SYBR Green (Roche) in a 20 µl reaction mix. The qPCR experiments were conducted in triplicate. Actin mRNA was used as an internal reference control.

### Proteasome Activity Assay

The chymotrypsin-like protease activity assay of the proteasome was performed by isolating the proteosomal pellet as described by Qiu and coworkers [31]. The resuspended proteasome pellet (100 µg) was incubated with 30 µg of each *in vitro* expressed and purified HcPro and mutant proteins as well as with 50 µM MG132 at 30°C in separate experiments. These were later assayed in 20 mM Tris buffer (pH 8.0) with 5 mM MgCl_2_, 1 mM DTT, using cleavage of 200 µM fluorogenic peptide Z-Leu-Leu-Val-Tyr-amido-methyl coumarin (AMC; Sigma) in the absence of ATP for 30 minutes after the addition of the substrate and stopped by addition of 1% sodium dodecyl sulfate (SDS) [32]. The released AMC was excited at 380 nm and fluorescence intensity was measured at 440 nm. Activity was calculated using an AMC standard curve made under the same conditions.

### Plasmid Constructs

We designated the papaya homologues of *A. thaliana* Atpaa (encoding the α1 subunit of the 20S proteasome) and Atpae (encoding the α5 subunit of the 20S proteasome, having RNase activity), as PAA and PAE, respectively, as previously described [14]. Details of all the primers used to develop different plasmid constructs are given in Table S1. The cDNAs encoding the papaya 20S proteasome subunits PAA and PAE were amplified by PCR using primers PAA1 F/PAA1 R and PAE1 F/PAE1 R, respectively, and were ligated into the pGEM-T vector for transformation of *Escherichia coli* strain DH5α. The nature of the clones was confirmed by sequencing. The clone of the HcPro gene was already available in our laboratory [33], [34].

For yeast two-hybrid assays, the full-length coding sequence of PRSV HcPro was amplified by PCR using the primers HcPro-EcoRI F/HcPro-PstI R and HcPro-BamHI R. The product was then ligated into pGBKT7 (BD) and pGADT7 (AD) following EcoRI/PstI and EcoRI/BamHI digestion, respectively. The full-length coding sequences of the proteasome subunits (PAA and PAE) were also ligated in both pBDKT7 and pGADT7 vectors with EcoRI/BamHI digestion after PCR amplification with PAA1 EcoR1 F/PAA1 BamH1 R and PAE1 EcoR1 F/PAE1 BamH1 R primers, respectively.

PRSV HcPro sequence was submitted to SMART-7 (http://smart.embl-heidelberg.de/), NCBI, for protein sequence analysis. Two mutant HcPro genes, designated HcPro-C35G-S35G (M1) and HcPro-KITC54-KITS54 (M2), were generated by PCR-mediated site-directed mutagenesis using primers KITC F/KITC R and CG F/CG R, respectively. All mutations were confirmed by sequencing. These mutated genes were also ligated in pGBKT7 and pGADT7 vectors using the same primer combination as for wt HcPro.

To develop the binary constructs of HcPro and its mutants, each was ligated into the plasmid pUC118, in the sense orientation, under the control of the cauliﬂower mosaic virus 35S RNA promoter (35SP) and transcriptional termination (35ST) sequences, using the HcPro Apa1 F and HcPro Xho1 R primers. A cassette of 35SP: Hc-Pro: 35ST sequence (2 kb) was ligated into the binary vector pCambia 2301 between the BamHI and HindIII sites. The binary construct of the PRSV coat protein (CP) gene was generated in pBI 121 using Coat Protein F and Coat Protein R primers.

The gene encoding the HcPro protein was ligated into pROK2-based BiFC vectors [35] to those corresponding to the split yellow fluorescent protein N- or C-halves (sYFPN- or sYFPC-) to generate pROK constructs sYFPN-HcPro, -HcPro (M1), and -HcPro (M2), as well as sYFPC-HcPro, -HcPro (M1), and -HcPro (M2), using the primers HcPro BamHI F and HcPro BamHI R. In a similar way, the construct sYFPC-PAA was developed after the PCR amplification of the gene using the primers PAA1 BamHI F and PAA1 BamHI R. The wt HcPro gene and the two mutants were ligated into the pMAL vector (pMAL protein expression and purification system, NEB), for the expression of proteins in a bacterial system, using the primers HcPro EcoRI F and HcPro PstI R.

### Yeast Two-Hybrid Assay

To determine the interaction of different pairs of proteins, individual protein-coding genes were ligated in the pGADT7 (AD) and pGBKT7 (BD) vectors and were co-transformed in *Saccharomyces cerevisiae* (strain AH109) using the EZ-Yeast transformation kit (MP Biomedicals). Transformants grown on auxotrophic double dropout media (SD/−leu−/trp) were confirmed by colony PCR using their respective primers. The co-transformed colonies from double dropout plates were streaked on quadruple dropout agar media (SD/−leu/−trp/−ade/−his+x-apha-gal). The colonies in which interactions of proteins of interest occurred were able to survive and grow on quadruple dropout agar media as blue-colored colonies as described in yeast protocol handbook (Clontech).

To confirm the expression of genes of interest, the expressed proteins were detected by western blotting using anti-c-myc antibodies with a dilution of 1∶1000 in PBS (Clontech) as described in the Yeast Protocol Handbook (ClonTech).

### Transient Expression of Genes in Plant Tissues

Cultures of *Agrobacterium tumefaciens* strain LBA4404 containing binary vectors were grown overnight to exponential phase in Luria Broth with their respective selective antibiotics, at 28°C. For infiltration, each bacterial culture harboring a different T-DNA was diluted to a final OD at 600 nm (OD600) of 0.2 with infiltration buffer (10 mM MES, 4 mM acetosyringone and 10 mM MgCl_2_). Different cultures harboring different T-DNAs were then combined and mixtures were infiltrated into fully expanded leaves of one month old *Nicotiana benthamiana* plants, using a syringe. The transcript expression levels of the infiltrated DNA were checked by qRT-PCR.

For BiFC studies, *Agrobacterium* cultures, each carrying a binary vector with the corresponding split yellow fluorescent protein N- or C-termini (sYFPN- or sYFPC-) fused to the proteins of interest, were co-infiltrated together into the same leaf tissue. Epidermal cells of *N. benthamiana*–infiltrated tissue were monitored for fluorescence from either green fluorescent proteins (GFP), or the reconstituted sYFP fluorophores of tagged proteins. Imaging was made with Leica SP1 and SP2 (Leica Microsystems, Heidelberg, Germany) confocal laser scanning microscopes, using fresh, non-treated leaf tissues and either water immersion or water dipping objectives, as described previously by [36].

For the agro-infiltration patch assays, an *Agrobacterium* culture expressing a *GFP* reporter gene from a pROK2 binary vector/PRSV-CP gene from a pCambia2301 was co infiltrated with *Agrobacterium* containing the binary vector pCambia2301 expressing genes encoding proteins (HcPro and its mutants) to be tested for RNA silencing activity. The transcript expression levels of the infiltrated DNA were checked by qRT-PCR using GFP F/GFP R primer set and 3′PRSV F/3′PRSV R primer set.

### Plant Protein Extraction and Western Blot Analysis

For protein extraction, 0.1 g of infiltrated tissue was ground to a powder in liquid nitrogen. Total plant proteins were then extracted with PBS and quantified by Bradford assay [37]. An equal volume of 2X Laemmli loading buffer, pH 6.8 (containing 1% 2-mercaptoethanol, and 1% SDS) was added before boiling and equal concentration of proteins (specific details mentioned in figure legends) were fractionated by 12% SDS-PAGE (10). Proteins in the gel were wet-blotted onto nitrocellulose membranes. For immunological detection of ubiquitin, a rabbit anti-ubiquitin polyclonal antiserum (Sigma) was used. Blotted proteins were detected using commercially available alkaline phosphatase-conjugated anti-rabbit secondary antibodies (Sigma) and Sigma Fast BICP/NBT substrate. GFP antibodies, with a dilution of 1∶500 in PBS (G-Biosciences) were used to detect GFP tagged HcPro and its mutant as well as in agro-infiltration patch assay. For detection of CP in-house raised polyclonal antibody were used.

### Expression of PRSV HC-Pro and its Mutants in Bacteria

To investigate the role of PRSV HcPro and its mutants in the RNA silencing pathway, the corresponding genes were ligated in the vector pMAL-c2X and expressed from a ‘tac’ promoter as a MBP-HcPro fusion protein in *E. coli* strain BL21 (New England Biolabs, Beverly, MA), as described [38]. The fusion proteins were grown in LB-rich medium containing ampicillin and 2% glucose followed by addition of IPTG (0.4 mM) and induction for 4 hours at 28°C. All subsequent steps were performed at 4°C. Cells were collected by low-speed centrifugation and resuspended in column buffer (20 mM Tris-HCl, pH 7.4, 200 mM NaCl, 1.0 mM EDTA and 10 mM β-mercaptoethanol). Affinity chromatography was performed as recommended by the manufacturer (New England Biolabs, Beverly, MA) after the sonication of the cells. The purified fusion proteins were analyzed by 12% SDS-PAGE.

### *In vitro* Small RNA Binding of PRSV HcPro and Gel-shift Mobility Assays

Double-stranded siRNA171 (siR171 and its complement) was formed by mixing equimolar amounts of chemically synthesized small single-stranded RNAs, boiling them, and cooling them to room temperature. For *in vitro* protein-RNA binding assays, 30 pmol of synthetic siRNAs were incubated with different concentrations of dialyzed protein(s) in 10 mM Tris-HCl, pH 7.5, 1 mM MgCl_2_, 1 mM dithiothreitol, and 50 mM NaCl. Protein-RNA complexes were analyzed in 2% agarose, Tris-acetate gels as described [39]. RNA bands were visualized by ethidium bromide staining and quantified using an Alpha-Imager version 6.0.

### *In silico* Analysis

Identification of PEST region in the protein sequences of PRSV was predicted by ePEST Find program (http://emboss.bioinformatics.nl/cgi-bin/emboss/epestfind). The sequences of the PRSV proteins were downloaded from NCBI database. The instability index of the viral proteins was calculated by the Protparam program (http://web.expasy.org/). The significance of the results obtained in ELISA and q-PCR experiments was analysed using SASink software (http://www.sas.com/technologies/analytics/statistics/). The significance level of the data was studied. One way Analysis of Variance (ANOVA) was used to compare the mean values.

## Results

### Inhibition of the Proteasome Helps Establish PRSV Infection

**Figure 1:**
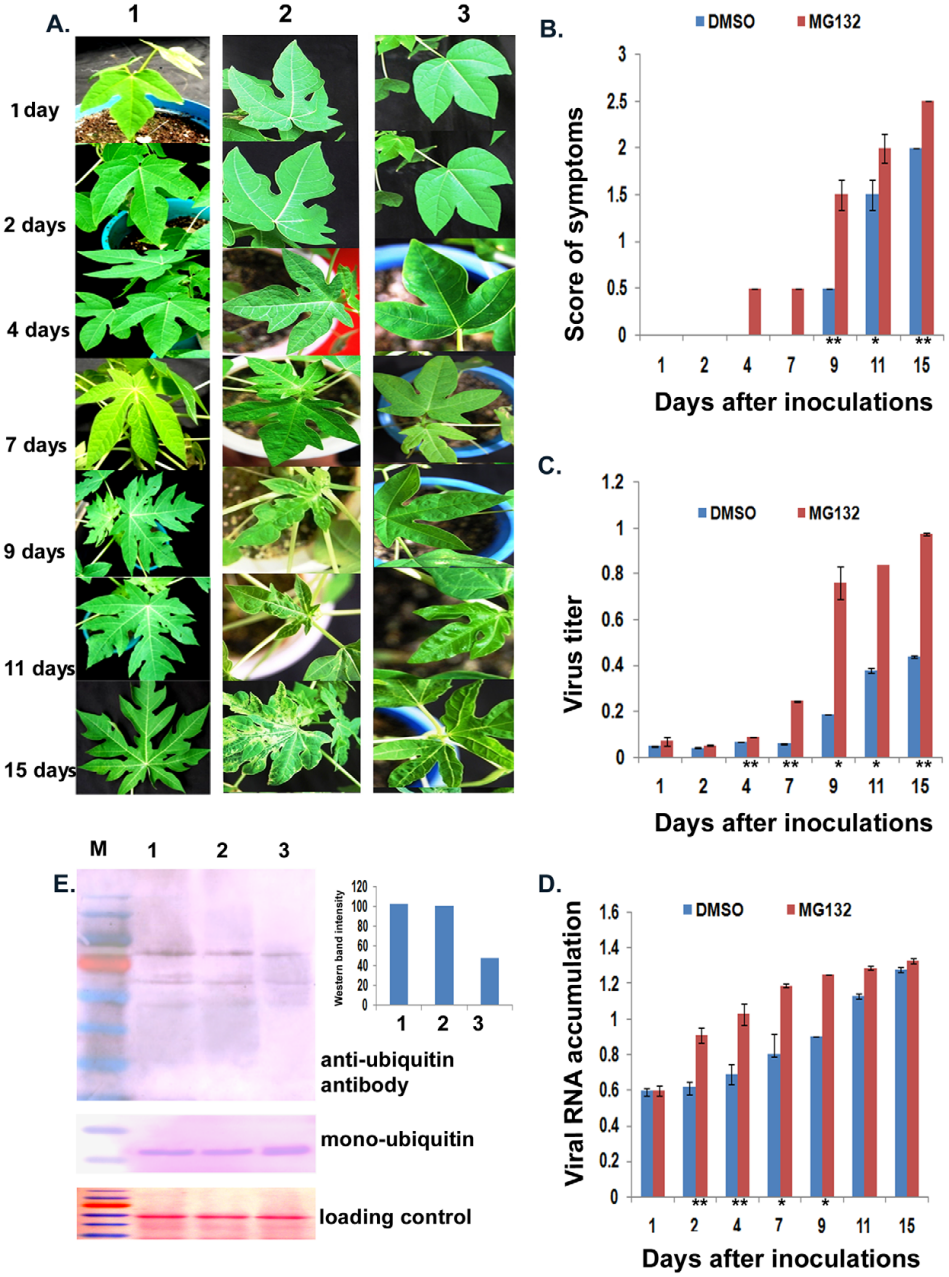
Proteasomal inhibitor MG132 effect on PRSV infection. A. Symptoms produced by PRSV after mechanical inoculation in both MG132 (2) and DMSO (3) treated papaya plants in comparison to control (1) MG132 treatment without virus inoculation, with time. Papaya seedlings were treated with MG132 (50 µM in 0.02% DMSO) and DMSO (0.02%). The first appearance of flecking symptom on papaya leaves was recorded. Mere treatment of MG132 without viral inoculation does not result in any kind of symptoms or leaf abnormality. B. Symptoms severity kinetics in PRSV-infected papaya leaves treated with DMSO or MG132 in DMSO. Symptom severity was scored by early appearance of flecking followed by mosaic development on leaves, which further resulted in prominent mosaic and chlorosis. Symptom severity score was rated on a 3-point scale: 0 = no symptoms, 0.5 = appearance of mid mosaic, 1 = mild mosaic and chlorosis without leaf deformation, 1.5 = chlorosis with appearance of leaf deformation, 2 = clear mosaic with slight leaf deformation, 2.5 = clear mosaic and chlorosis with slight leaf deformation, 3 = strong mosaic all over the leaflets with leaf deformation. C. Virus accumulation kinetics in PRSV-infected papaya leaves treated with DMSO or MG132 in DMSO. Virus titer was estimated at different time intervals post viral inoculation by DAC-ELISA using PRSV polyclonal antibodies (dilution 1∶1000). D. Relative qPCR quantification of PRSV RNA with amplification of 3′ end of the genome overlapping CP encoding region in infected plants. Quantitative estimation of viral transcript using SYBR Green in quantitative PCR; amplifying 3′ region of viral genome (nucleotide 9389 to 9566). Error bars represent standard deviation of mean data in three repeat experiments. Asterisks indicate statistically significant difference (* P<0.001 and ** P<0.005 by one way ANOVA). E. Western blot of proteins extracted from papaya leaves treated with DMSO prior to inoculation (3) and MG132 in DMSO from inoculated (2) and uninoculated plants (1). Total protein was isolated from leaves after 24 hours post treatment. Ten µg of protein from each treatment was fractionated in a 12% SDS-PAGE and western blotting was done using a rabbit polyclonal anti-ubiquitin antiserum. A range of proteins from 26 to 172 kDa was detected by immunoblotting. Immunoblot detection of free ubiquitin was done with 5 µg of protein in a 14% SDS-PAGE. MG132 treatment resulted in accumulation of proteins; as shown by the western blot and by a graph showing relative band intensities.

Since the 20S proteasome has been shown to affect viral RNA accumulation [12], [15], we sought to assess whether inhibition of the 20S proteasome would result in an increase in virus accumulation. Therefore, papaya leaves were treated with MG132 in DMSO or DMSO alone, and were then inoculated mechanically with PRSV, 2 hours after infiltration. The first visible symptoms of virus infection in MG132/DMSO-treated plants were observed as early as 4 days post inoculation (dpi) [Figures 1, A (2) and B] as compared to DMSO-treated plants, that exhibited the first sign of symptoms at 9 dpi followed by the advancement of symptom severity (Figures 1, A (3) and B); the normal occurrence of symptoms of PRSV infection after mechanical inoculation takes as long as 9 to 15 dpi [40]. No symptom-like abnormalities appeared due to MG132 treatment without virus inoculation [Figure 1A (1)]. The virus titers were quantified from the samples collected at various dpi from both MG132-treated and control DMSO-treated plants. From 4 dpi onwards, the accumulation of virus in MG132-treated plants was always more than that of the control DMSO-treated samples (Figure 1C). In addition, qRT-PCR experiments were done using RNA from the same infected plants to estimate the viral RNA levels. The increase in viral RNA levels was almost 1.5-fold for MG132-treated vs. control DMSO-treated plants from 2 to 9 dpi. However, by 15 dpi, there was not much difference in accumulation of viral RNA between the two types of samples (Figure 1D).

The patterns of total ubiquitinated protein accumulation in MG132-treated and PRSV inoculated vs. uninoculated plants was compared with those in the DMSO-treated plants by western blot analysis using rabbit, polyclonal anti-ubiquitin antiserum. The presence of relatively higher amounts of ubiquitinated proteins in MG132-treated leaves compared to DMSO-treated leaves was observed (Figure 1E).

### PRSV HcPro Exhibits Differential Interaction with the 20S Proteasome Components

**Figure 2:**
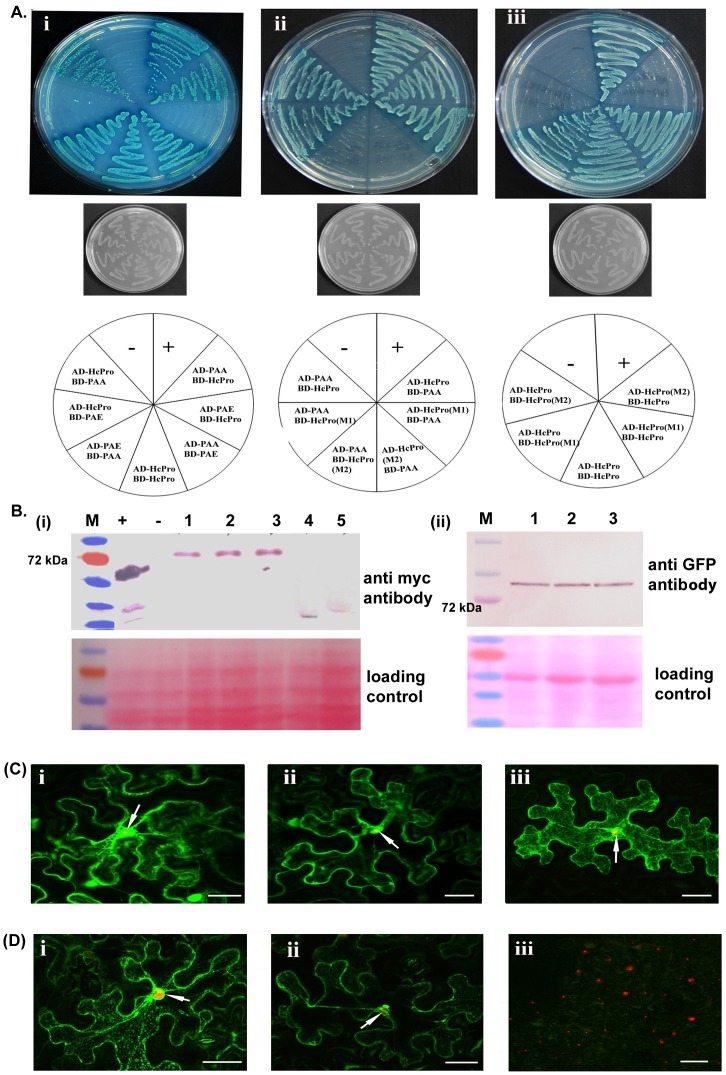
PRSV HcPro interactions and localization. A. For yeast two-hybrid assays the constructs were transformed in *Saccharomyces cerevisiae* strain AH109 cells. (Lane 1) The cells were grown on SD/−Ade/−His/−Leu/−Trp (quadrauple drop out)+X-α-gal media, streaked from the cells grown on double drop out media. pGBKT7-p53/pGADT7-RecT (positive control) symbolized as +, pGBKT7-p53−/pGADT7–lamin (negative control) symbolized as −, (Lane 2) the cells were grown on SD/−Leu/−Trp (double drop out media). Schematic representation of interactions is shown below the plate format where pGADKT7 is denoted as AD and pGBKT7 is denoted as BD. (i) Interaction of PRSV HcPro with the papaya 20S proteasome subunits PAA and PAE, and with itself. HcPro can interact with PAA but not with the PAE subunit of the proteasomal complex and HcPro exhibit a self-interaction. (ii) Interaction of PRSV HcPro mutants with papaya proteasome subunit PAA. HcPro (M1) can interact with PAA, but HcPro (M2) cannot interact with PAA. (iii) Interaction of PRSV wild type (wt) HcPro with itself and with the HcPro mutants. The wt HcPro can interact with itself and with HcPro (M1), but not with HcPro (M2). B. (i)Western blot analysis of yeast total protein fractionated in a 10% SDS-PAGE and probed with mouse anti-myc antiserum for expression of all the analyzed HcPro mutants and proteasomal proteins, along with the positive control; pGBKT7-p53/pGADT7-RecT and negative control (protein isolated from non-transformed yeast cells). (ii) Western blotting of total plant protein after agroinfiltration of GFP-tagged HcPro and its mutants. The total proteins were fractionated in a 12% SDS-PAGE and blotted onto a nitrocellulose membrane. The western blot probed with polyclonal GFP antibody represents the accumulation of the wt and mutant HcPro in cellular conditions. C. Subcellular distribution in *Nicotiana benthamiana* epidermal cells of green fluorescent protein (GFP)-tagged PRSV HcPro and its mutant proteins M1 and M2, expressed transiently by agroinfiltration, as well as of fibrillarin tagged with monomeric red fluorescent protein (Fib-mRFP), as a nucleolar marker. Green fluorescence derived from proteins tagged at their C-terminus with GFP: (i) HcPro-GFP, (ii) HcPro (M1)-GFP, or (iii) HcPro (M2)-GFP. In all three cases, fluorescence was found in both the cytoplasm and nucleus. Red fluorescence derived from Fib-mRFP was confined to the nucleolus (arrows). Bars in the lower right corners represent 20 µm. D. Bimolecular fluorescence complementation (BiFC) between PRSV HcPro and *Carica papaya* PAA protein tagged at their N- and C- termini, respectively, with split yellow fluorescent protein halves (sYFP) expressed transiently by agroinfiltration in *Nicotiana benthamiana* epidermal cells: (i) sYFPN-HcPro plus sYFPC-PAA, (ii) sYFPN-HcPro (M1) plus sYFPC-PAA, and (iii) sYFPN-HcPro (M2) plus sYFPC-PAA. In this last case, a field of several epidermal cells is shown instead of a single cell, with nucleoli appearing red (arrows) because of the presence of Fib-mRFP. The interactions of HcPro and HcPro (M1) with the PAA proteasomal subunit both took place in the cytoplasm, with fluorescence distributing mainly with a reticulate appearance. Bars in lower right corners represent 20 µm in all panels, except in the lower panel to the right, where it represents 50 µm.

In the yeast two-hybrid assay, co-transformed colonies expressing the PAA subunit of the 20S proteasome and HcPro showed interaction, as shown by their ability to produce blue colonies on selection plates for SD/−leu/−trp/−ade/−his+X-α-gal [Figure 2A(i)], regardless of whether the HcPro and PAA were fused to the activation or binding domains. By contrast, in co-transformed colonies expressing the PAE subunit of the 20S proteasome there was no interaction with HcPro [Figure 2A(i)]. On the other hand, the PAE and PAA subunits of the 20S proteasome were able to interact with each other [Figure 2A(i)]. The yeast two-hybrid assays also confirmed that the wt PRSV HcPro protein could interacts with itself *in vivo* [Figure 2A(i)].

The interaction between the PVY HcPro and the *A. thaliana* PAA subunit was shown to involve the N-terminal region (amino acids 1–97) of the HcPro [14]. To delimit the motif (near the N-terminus) involved in the interaction between the PRSV HcPro and the papaya PAA subunit, motif characterization studies were done using the SMART program. These showed the presence of several zinc finger-like motifs in the N-terminal domain of PRSV HcPro (Table 1): e.g., ZnF_AN1, mostly present in stress-associated proteins and with a role in regulating immune responses (located at amino acids 2–31); ZnF_UBR1, a putative zinc finger in N-recognin, a recognition component of the N-end rule pathway (amino acids 33–79); an overlapping domain, ZnF_NFX, with a role in repression of transcription (amino acids 35–59); and ZnF_ZZ, with a role in protein-protein interactions (amino acids 48–86). A RING-domain, signature-element of ubiquitin E3 ligase also was predicted in the region bordered by HcPro amino acids 32–78 (Table 1). Interestingly, there are only two conserved cysteine residues in the form of CG35 and KITC54, respectively, in this particular region of the HcPro amino acid sequence (Figure S1A). Therefore, to disrupt the disulfide bond forming potential of the amino acid cysteine, we generated two mutants in the N-terminal region, altering either C35G to S35G or KITC54 to KITS54, designated as HcPro (M1) and HcPro (M2), respectively (Figure S1B).

**Table 1:** *In silico* analysis of protein motifs present in PRSV-HcPro using SMART.

Name	Begin	End	E-value	Reason
ZnF_AN1	2	31	6.73e+02	Threshold
FYVE	21	63	8.37e+02	Threshold
AWS	22	65	2.79e+03	Threshold
*RING	32	78	1.43 e+03	Threshold
ZnF_UBR1	33	79	9.92 e+02	Threshold
Zpr1	33	138	1.67 e+03	Threshold
ZnF_NFX	35	59	1.60 e+03	Threshold
LRR_CC	38	63	2.82 e+02	Threshold
CGGC	43	132	4.93 e+04	Threshold
*ZnF_ZZ	48	86	3.62 e+02	Threshold
Ami_2	50	193	2.19 e+03	Threshold
Arfaptin	57	276	7.56 e+04	Threshold
PCRF	57	127	1.59 e+05	Threshold
SRP54_N	58	123	7.00 e+04	Threshold
SPEC	67	173	1.36 e+03	Threshold
L27	76	129	2.11 e+03	Threshold
GatB_Yqey	79	200	8.93 e+04	Threshold
B12-binding_2	95	170	1.59 e+05	Threshold
IL4_13	97	223	6.17 e+02	Threshold
UTRA	100	219	1.48 e+05	Threshold
Low complexity	116	127	–	Overlap
SprT	143	255	3.78 e+03	Threshold
BHD_1	152	219	5.73 e+04	Threshold
Bro-N	278	373	1.60 e+05	Threshold
Skp1	322	393	1.63 e+05	Threshold

* Presence of important motifs like RING and ZnF_ZZ at the N-teminal region of the protein.

The mutants HcPro (M1) and HcPro (M2) were expressed in shuttle vectors in yeast. Impairment of HcPro interaction with the PAA subunit of the papaya 20S proteasome in the yeast two-hybrid assay was observed for HcPro (M2), but not for HcPro (M1), regardless of the orientation of the two proteins [Figure 2A(ii)]. Interestingly the mutant HcPro (M2) also failed to interact with the wt HcPro [Figure 2A (iii)], although the HcPro (M1) and wt HcPro were able to interact with each other [Figure 2A(iii)]. Similar levels of expression of the mutant and wt HcPro proteins were detected by western blot [Figure 2B (i)].

The mutants HcPro (M1) and HcPro (M2) also were expressed from a binary vector in *N. benthamiana* plants, either as fusions to GFP, or as fusions with split YFP. Using confocal microscopy, imaging of GFP fused to the C-terminus of HcPro and its mutants (M1 and M2) was done. These proteins were found to be distributed in both the nucleus and the cytoplasm, when expressed by agroinfiltration in *N. benthamiana* leaves [Figure 2C (i, ii and iii)], although HcPro (M2) also was distributed in a reticulated pattern across the cytoplasm. Comparable levels of accumulation of wt and mutant HcPro proteins in plant cells were shown by an immunoblot assay with GFP antibodies [Figure 2B(ii)]. The split YFP fusions of the wt and mutant HcPro proteins were co-expressed with PAA by agroinfiltration of *N. benthamiana* leaves. The interactions of the split YFP-fused proteins inside the plant cells were detected by BiFC. The results visualized the interaction of wt HcPro with the PAA subunit of the 20S proteasome in the plant [Figure 2D(i)], similar to that of its mutant M1 [Figure 2D(ii)], although mutant M2 failed to do so [Figure 2D(iii)]. Furthermore, complexes between the PAA and either the wt HcPro or HcPro (M1) were found associated with the cytoplasm and its trans-vacuolar strands (Figure 2D).

### PRSV HcPro Interferes with the Protease and RNase Activity of 20S Proteasome

**Figure 3:**
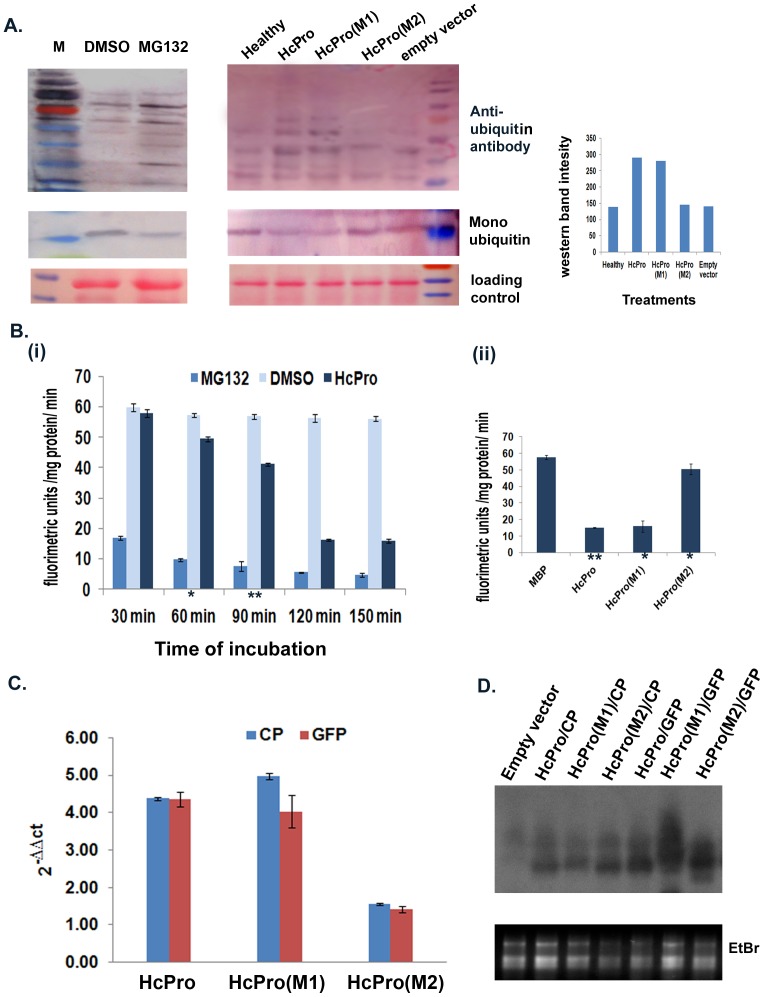
HcPro effects on 20S proteasome catalytic activities. A. Quantification by western blotting of the steady-state levels of accumulation of total ubiquitinated protein in the infiltrated patch using a rabbit polyclonal anti-ubiquitin antiserum. The total plant protein (10 µg) was fractionated in a 12% SDS-PAGE in each case. A range of proteins from 17 to 130 kDa was detected by immunoblotting. Immunoblot detection of free ubiquitin was done with 5 µg of protein in a 14% SDS-PAGE. The lower panel shows a Ponceau S stained membrane. The right panel shows the graph depicting relative band intensities of HcPro, HcPro (M1) and HcPro(M2). B. (i) Proteasome activity assay of HcPro along with MG132 after different time of incubations. The assay was started by addition of fluorogenic substrate after incubation of 100 µg of proteosomal pellet with 30 µg HcPro without ATP. (ii) Comparison of proteasome inhibitory action of wt HcPro with its mutants after 120 minutes of incubation. The MBP was taken as control. The wt HcPro and HcPro (M1) showed a similar trend whereas HcPro could not inhibit the proteasomal protease function after 120 minutes of incubation. All the data are means ±SD of three repeat assays. Asterisks indicate statistically significant difference (* P<0.001 and ** P<0.005 by one way ANOVA). C. Relative expression levels of PRSV CP and GFP mRNA estimated by qPCR using the 2^−ΔΔCt^method. The accumulation of RNAs in *N.benthamiana* leaves was compared from leaf samples co-infiltrated with *Agrobacterium* harboring a binary plasmid (empty vector) along with binary vectors expressing either the PRSV CP or GFP genes, with leaf samples co-infiltrated with *Agrobacterium* harboring a plasmid expressing wt HcPro, HcPro (M1) or HcPro (M2) along with plasmids expressing either the PRSV CP or GFP genes. The actin mRNA level was used as an internal standard. The data represented are means of three independent experiments in each case. The error bars represents deviation observed in three repeat assays. Asterisks indicate statistically significant differences (* P<0.001 and ** P<0.005 by one way anova) D. Detection of HcPro RNA transcript by RNA gel blot. The total RNAs extracted from the various CP and GFP expressed samples were analyzed by agarose gel electrophoresis and blotted onto nylon membrane. The blot was hybridized by radioactive HcPro probes.

To investigate the consequences of HcPro-PAA interaction on the protease activity of the proteasome, the amount of total ubiquitinated protein was estimated from the *N. benthamiana* leaves infiltrated with *Agrobacterium* cultures containing binary constructs expressing HcPro or its two mutant variants. The levels of ubiquitinated protein expression were compared with those in MG132-infiltrated leaves. Western blot analysis showed a rise in the amount of ubiquitinated proteins in leaves infiltrated with HcPro when compared to leaves infiltrated with the control empty vector (pCambia2301). The mutant HcPro (M1) also increased the level of the total of ubiquitinated proteins, comparable with the wt HcPro, whereas the mutant HcPro (M2) failed to do so (Figure 3A). The corresponding increase in the accumulation of ubiquitinated protein both in wt HcPro and MG132 treatments, with respect to their controls, indicates that HcPro mimics the response to MG132 treatment.

### HcPro Affects 20S Proteasome *In vitro* Activity Assay

The crude proteasome extract was assayed for the chymotrypsin like activity in the presence of Z-Leu-Leu-Val-Tyr-amido-methyl coumarin peptide which is a specific substrate for 20S proteasome. The reaction was carried out in the absence of ATP to rule out the 26S proteasomal activity on the fluorogenic substrate. MG132 was used as the positive control for the experiment. The reaction was carried out at different time intervals. Proteasome extracts were incubated for 30 min to 150 min with wt HcPro and its mutants expressed and purified from bacteria before starting the activity assay by addition of fluorogenic substrate [Figure 3B (i)]. There was a decrease in the protease activity of the proteasome when incubated with MG132 from 30 min (incubation period) and HcPro protein from 60 min (incubation period) onwards, which became static after 120 min. Based on this observation, subsequent assays involving HcPro mutants were carried with 120 minutes incubation period. HcPro (M1) followed the same decreasing pattern in protease activity, but HcPro (M2) did not [Figure 3B(ii)]. Although the MG132-mediated inhibition of the 20S proteasome was prominent at 30 min of incubation, HcPro could reflect a similar inhibitory pattern on the 20S proteasomal activity at later stages of *in vitro* incubation. The mutation in the KITC54 motif at the N-terminus of the HcPro protein negatively affected this inhibition as evident by the fluorometric reading in the *in vitro* assay.

**Figure 4:**
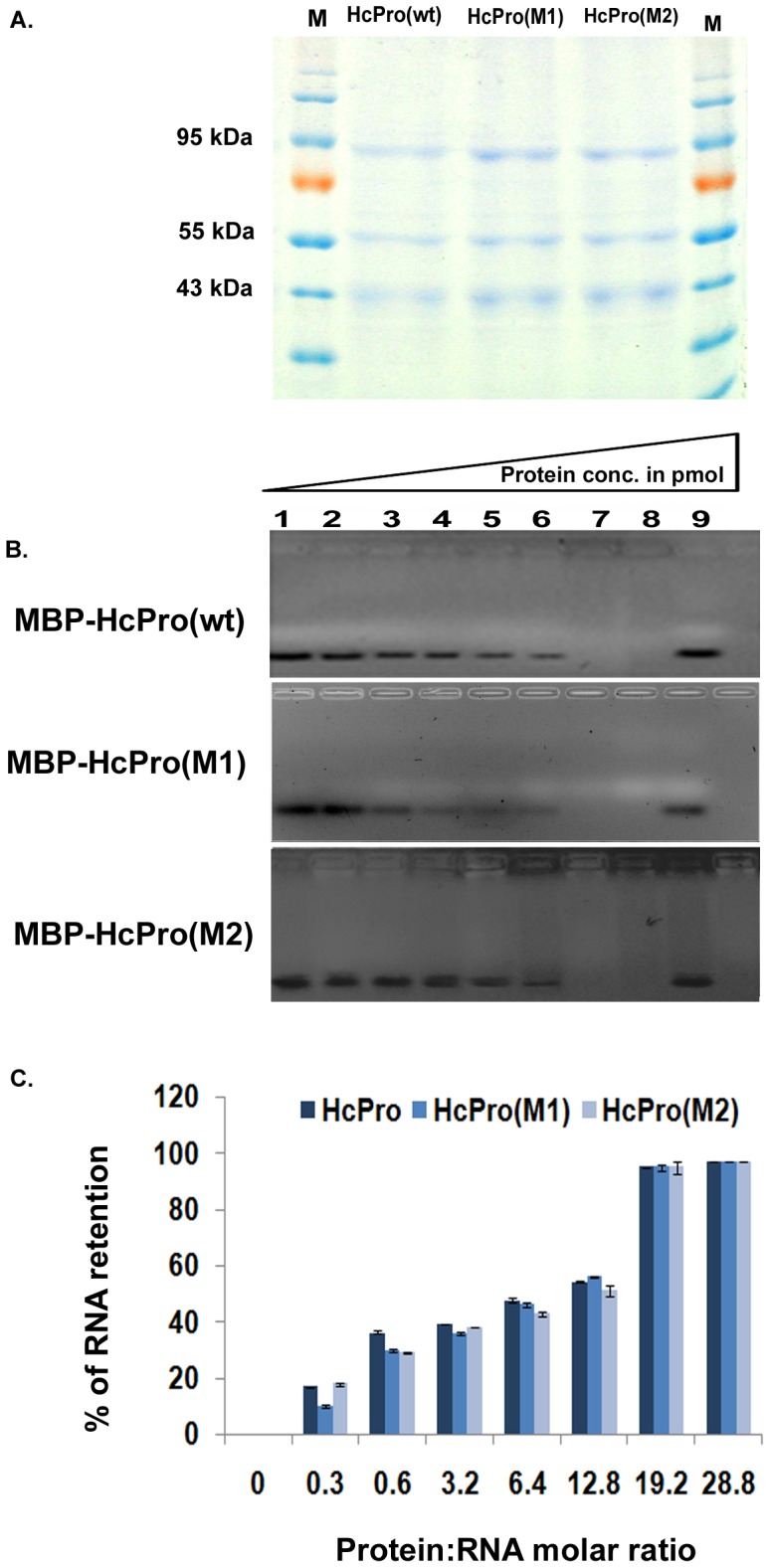
Small RNA binding ability of HcPro and its mutants. Electrophoretic mobility shift assay for wild-type (wt) HcPro and its mutants. A. Purified preparations of HcPro and mutant proteins on Coomassie-stained 12% SDS-PAGE purified from *Escherichia coli* BL21 cells. MBP-HcPro/mutants fusion protein (92.5 kDa) B. Electrophoretic mobility shift assay for HcPro and its mutants. Different concentrations (lane 2–8) of purified HcPro or its mutants were incubated with 30 pmol of synthetic double stranded small RNA (siRNA171) for 30 minutes at 25°C. Lane 1, 30 pmol of synthetic, double-stranded small RNAs (siRNA171) without any protein added; lane 9, 30 pmol of synthetic, double-stranded small RNAs (siRNA171) incubated with MBP for 30 minutes as control. C. Graphical representation of the RNA binding results for the wt HcPro and its mutants. % RNA retention (determined by band intensities) was plotted against protein:RNA molar ratios in the binding assays of HcPro and its mutants with double stranded siRNA171. The data are means ±SD of four repeat assays. The significance level of the data are shown by asterisks (* P<0.001 and ** P<0.005).

To further investigate the role of HcPro in affecting the RNase activity of the 20S proteasome, two genes, one viral (*PRSV CP*) and one non-viral (*GFP*), were co-expressed transiently in *N. benthamiana* leaves, together with PRSV HcPro, the HcPro mutants M1 and M2, or the empty binary vector. The transcript accumulation for CP and GFP was monitored by qRT-PCR (Figure 3C), while the presence of wt and mutant HcPro RNA transcripts was shown by RNA gel blot analysis (Figure 3D). Both the CP and GFP RNA transcript levels were increased 4- to 5-fold by either wt HcPro or HcPro (M1), but less than 2-fold by HcPro (M2), with regard to the values in co-infiltration experiments with the empty vector (Figure 3C). The increase in the accumulation of transcripts might be due to the involvement of HcPro in the suppression of the RNA silencing intrinsic pathway, and/or by affecting the RNase activity of the proteasome, although HcPro (M2) did not respond in a similar way. The noticeable lack of increase in RNA transcript (GFP) accumulation in the case of HcPro (M2) could be attributed to the impairment of either the small RNA-binding activity of HcPro (M2)/altered RNA silencing suppression activity or its lack of interaction with the proteasomal subunit PAA. However, previous mutational analyses done using the HcPro of both tobacco etch virus and zucchini yellows mosaic virus indicated that HcPro sequences involved in small RNA binding and affecting RNA silencing suppression were not located in the N-terminal region of the HcPro [41]–[43]. To analyze this further, a small RNA binding assay was performed using MBP-fused HcPro and its mutant proteins (92.5 kDa), purified after bacterial expression (Figure 4A). Increasing concentrations of purified HcPro and its two mutants, M1 and M2, were assayed for their binding ability to 30 pmol of a synthetic double-stranded silencing-inducing (si) RNA (Figure 4B). The complete binding of 30 pmol of siRNA was achieved using 577 pmol of wt HcPro protein. The same binding profile was obtained using HcPro mutants M1 and M2, indicating that the HcPro mutations did not affect the siRNA binding capacity (Figure 4, B and C).

The activity of wt HcPro and its mutants in in RNA silencing suppression was also studied in an agroinfiltration patch assay [36] in *N. benthamiana* leaves. There was no appreciable increase in the GFP protein levels during transient expression of wt HcPro, M1, or M2 when compared to the control p19 from tomato bushy stunt virus (data not shown). HcPro and its mutants showed differential accumulation of GFP transcript levels, although their small RNA binding capacity and RNAi silencing activity remains same. These observations probably suggest that accumulation of GFP transcript in presence of HcPro might be attributed for its ability to modulate RNase activity of proteasome and not because of its role in RNA silencing. The intrinsic RNA silencing activity of the host might be responsible in non-achieving the corresponding increase in GFP protein proportionate to GFP transcript.

## Discussion

### Proteasome Inhibitor MG132 Facilitates Virus Accumulation

Our results showed that inhibition of the proteasome in papaya by MG132 enhanced the accumulation of PRSV, which also was reflected in the faster symptom development, as well as enhanced viral RNA accumulation beyond 4 dpi (Figure 1). There are two different catalytic activities associated with the 20S proteasome that could play such a role in affecting PRSV accumulation in papaya: first, protease activity [7], [10]; and second, the associated RNase activity [9]. The accumulation of PRSV by proteasomal inhibition with MG312 might be due to either the role of the 20S proteasome in altering the intrinsic RNA silencing pathway, or a change in its protease and/or RNase catalytic activity. If the 20S proteasome degrades an RNA silencing pathway protein of the host, such as Argonaute, it might interfere in the plant defense, by suppressing the RNA silencing pathway. In such a scenario, the impairment of the 20S proteasome by MG132 would lead to lower virus titers and would make the plant more resistant, as shown in the case of PVX [11] and for poleroviruses [43]. By contrast, knocking out the 20S proteasome component led to higher virus accumulation [15].

Viral accumulation is directly related to the number of virus multiplication cycles, where one or more viral proteins play an important role. When the *in silico* analysis for comparative stability of the viral proteins towards ubiquitin-proteasome mediated degradation were studied for ten proteins of PRSV, only the P1 protein of PRSV was found to be prone to ubiquitin-proteasome degradation. Nine other viral proteins including HcPro, CP and proteins involved in replication, were found to be stable (Table S2). These observations suggest that the inhibition of the proteasome facilitates virus accumulation, predominantly through increased accumulation of viral RNAs in the initial stages of the infection cycle. An early establishment of virus during proteosomal inhibition may be a result of accumulated *de novo*-synthesized viral RNAs and hence translated proteins, important for the virus multiplication cycle. This could then further results in earlier and more severe symptom development, which supports a role for the 20S proteasome in an essential antiviral defense mechanism of the host plant.

### PRSV HcPro Interacts with the PAA Subunit of the 20S Proteasome

Based on our initial observation regarding PRSV accumulation in papaya during proteasomal inhibition, as well as the earlier, albeit contradictory, observations that *A. thaliana* proteasome subunits interact with the HcPro of two other potyviruses [14], [15], we assessed the probable involvement of the PRSV multifunctional HcPro protein in modulating the 20S proteasome activities. We have demonstrated that the PRSV HcPro was able to interact with the PAA subunit, but not with the PAE subunit of the 20S proteasome from papaya, even though the latter mediates the RNase activity of the 20S proteasome (Figure 2). This inconsistency could be mitigated by our observation of an interaction between the PAA and PAE subunits. Thus, interaction of HcPro with the PAA subunit may either be sufficient to impede the function of the associated PAE (RNase) subunit, as suggested previously by Jin et al. [14] for PVY, or prevent the interaction of the PAA and PAE subunits. Our results are in accordance with the previous observation [14], in which the PVY HcPro was able to interact with the *A. thaliana* PAA but not with the *A. thaliana* PAE. By contrast, the LMV HcPro showed direct interaction with the *A. thaliana* PAE [15]. It is conceivable that the HcPro of different potyviruses interacts with different components of the 20S proteasome, depending on specific virus-host combinations, and might also lead to subcellular re-localization of these proteins, at least in part (Figure 2D).

A previous study [14] also showed that the N-terminal region (amino acids 1–97) of the PVY HcPro was necessary for the interaction with the proteasomal subunit PAA. To define further the probable domain at the N terminal region of the HcPro involved in this interaction, an *in silico* analysis was done by ClustalW multiple alignment. Based on this analysis, two domains, CG35 and KITC54, were found to be conserved among the HcPro proteins of a range of potyviruses. To date no function has been assigned to the CG35 domain whereas the KITC54 domain was found to be involved in aphid transmission of the virus [23], [44], [45]. Using the SMART program (Table 1), the KITC54 domain was also found to overlap with a predicted RING motif (32–78 aa), a signature element of ubiquitin E3 ligase, along with ZFN_ZZ (48–86 aa), a motif important for protein-protein interactions. These predicted domains might play some role in modulating UPS function beside its role in interaction with 20S core proteasomal components, which needs to be investigated further. Previous studies [44]–[46] also suggested the importance of this cysteine-rich region located in the N-terminal region of HcPro in self-interaction, because of its homology with zinc finger-like motifs. Based on these results, we can assign a novel function to the conserved HcPro KITC54 domain for the interaction with the 20S proteasome PAA component of the host, besides having a role in aphid transmission.

### PRSV HcPro Modulates Proteasomal Catalytic Activities

To investigate the consequences of HcPro-PAA interaction on the protease activity of the proteasome, the amount of total ubiquitinated protein was examined by an immunoblot assay of proteins extracted from *N. benthamiana* leaves infiltrated with *Agrobacterium* culture containing binary construct expressing HcPro or its mutants M1 and M2. The accumulation profile of total ubiquinated protein, after either MG132 treatment or expression of HcPro, suggests that HcPro mimics the proteasomal inhibitor’s mode of action *in vivo*, although HcPro (M2) failed to do so (Figure 3A). This result was further confirmed by in vitro assay using a 20S proteasome specific fluorogenic substrate. Our result is not in agreement with a previous finding, in which a slight stimulation of protease activity of the proteasome was observed in the presence of LMV HcPro, *in vitro* [12]. Interestingly, in the case of animal viruses, several reports have confirmed the inhibition by viral proteins of both protease activity and assembly of the 20S proteasome [27]–[29]. To date, it is not clear how the interaction between HcPro with the PAA subunit of proteasome affects the protease activity.

Ballut and his coworkers [9] first reported the association of an RNase activity with the 20S proteasome. They also demonstrated that the RNase activity of the 20S proteasome affected the accumulation of both TMV and LMV viral RNA. Dielen and his group [15] demonstrated the interaction of LMV HcPro with the *A. thaliana* PAE, while *in vitro* studies confirmed the involvement of the *A. thaliana* PAE in the RNase activity. Our results showed that transiently expressed PRSV HcPro and HcPro (M1) increased the accumulation of viral and non-viral exogenous RNAs, while HcPro (M2), which was impaired in its interaction with PAA, did not stimulate the accumulation of the exogenous RNAs (Figure 3C). These alterations in RNA accumulation could be caused by HcPro-mediated modulation of RNase activity associated with the 20S proteasome and/or through its suppression of RNA silencing pathways [38], [47]. HcPro and its mutants showed affinity for siRNA binding. The results of our siRNA binding assays suggest that mutation in HcPro at C35G (M1) and KITC54 (M2) did not affect its small RNA binding ability *in vitro* (Figure 4). These results suggest that the differential accumulation of exogenous RNAs was not due to the involvement of HcPro/mutants in the RNA silencing pathway, but by modulating the 20S proteasome RNase activity instead. This is in agreement with the earlier hypothesis [14] indicating that the PVY HcPro may indirectly inhibit the endonuclease activity of the 20S proteasome by binding to the PAA.

Based on our results we propose that HcPro mimics proteasome inhibitors in modulating the catalytic activity of the 20S proteasome, by interacting with its subunit through the KITC54 motif. These modulations may result in the increased viral titer after virus infection. However, further efforts are clearly required to unravel the precise role of these interactions.

## Supporting Information

Figure S1 **Conserved domain/motifs among potyviral HcPro (N-terminal) and mutational map of PRSV HcPro.** A. Schematic representation of the amino acid sequence in the N-terminal region of different Potyviral-HcPro sequences available in the database compared with PRSV-HcPro proteins. Different colors represent different amino acids. Conserved amino acids are highlighted in dark rectangles. The conserved cysteine-rich domains are shown in red rectangular boxes. B. Location of HcPro, HcPro (M1) and HcPro (M2) mutations in the HcPro coding sequence and the corresponding amino acid changes. The wild-type HcPro is given as wt HcPro.
(TIF)

Table S1 **List of primers used in this study.**
(DOC)

Table S2 ***In silico*** **analysis of PRSV protein sequences to evaluate the stability of the protein based on the N-end rule and presence of PEST sequence.**
(DOC)
