# Supplementary figures and images for: Inhibition of the Host Proteasome Facilitates Papaya Ringspot Virus Accumulation and Proteosomal Catalytic Activity Is Modulated by Viral Factor HcPro
Source: PLoS One. 2012 Dec 27;7(12):e52546. doi: 10.1371/journal.pone.0052546 (PMC3531422; doi:10.1371/journal.pone.0052546)

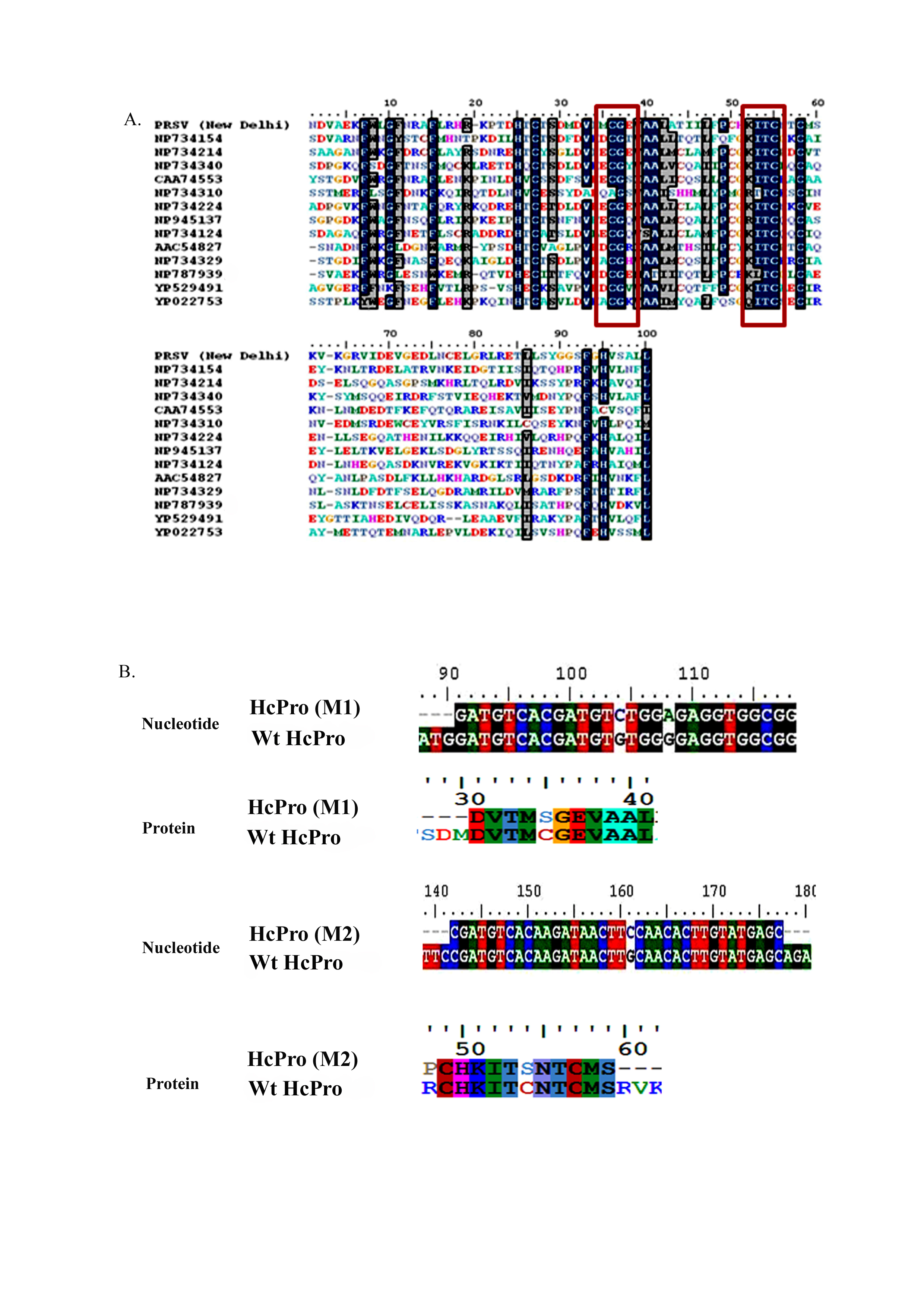

Supplement: Figure S1 — Conserved domain/motifs among potyviral HcPro (N-terminal) and mutational map of PRSV HcPro. A. Schematic representation of the amino acid sequence in the N-terminal region of different Potyviral-HcPro sequences available in the database compared with PRSV-HcPro proteins. Different colors represent different amino acids. Conserved amino acids are highlighted in dark rectangles. The conserved cysteine-rich domains are shown in red rectangular boxes. B. Location of HcPro, HcPro (M1) and HcPro (M2) mutations in the HcPro coding sequence and the corresponding amino acid changes. The wild-type HcPro is given as wt HcPro. (TIF) [file pone.0052546.s001.tif]
