# Supplementary material for: Inhibition of the Host Proteasome Facilitates Papaya Ringspot Virus Accumulation and Proteosomal Catalytic Activity Is Modulated by Viral Factor HcPro
Source: PLoS One. 2012 Dec 27;7(12):e52546. doi: 10.1371/journal.pone.0052546 (PMC3531422; doi:10.1371/journal.pone.0052546)
Supplement: Table S1 — List of primers used in this study. (DOC) [file pone.0052546.s002.doc]

| **S. No.** | **Name** | **Sequence** |
| --- | --- | --- |
|  | HcPro BamHI F | GAT GGA TCC AAT GAT GTG GCT GAA AAA TTC TG |
|  | HcPro BamHI R | GCT GGA TCC TCA CCC ACA ATG TAG TGC TTC AAT |
|  | PAA1 EcoRI F | GAT GAA TTC ATG AGT AGA GGA AGC GGA G |
|  | PAA1 BamHI F | GAT GGA TCC ATG AGT AGA GGA AGC GGA G |
|  | PAA1 BamHI R | GCT GGA TCC TCA GTC TCG TTC ACT AAT GG |
|  | PAE1 F | ATGTTTCTCACTAGAACTGAG |
|  | PAE1 R | TCAAAGACGAGCGATGACTG |
|  | PAA1 F | GAAGAAGATGAGTAGAGGAAG |
|  | PAA1 R | GATCAGTCTCGTTCACTAATG |
|  | CG F | GATGTCACGATGTCTGGAGAGGTGGCGGCTCTTGC |
|  | CG R | GCAAGAGCCGCCACCTCTCCAGACATCGTGACATC |
|  | KITC F | CCATGTCACAAGATAACTTCCAACACTTGCATGAGC |
|  | KITC R | GGTACAGTGTTCTATTGAAGGTTGTGAACGTACTCG |
|  | PAE1 EcoRI F | CCG GAA TTC ATG TTT CTC ACT AGA ACT GAG |
|  | PAE1 BamHI R | CCG GGA TCC TCA AAG ACG AGC GAT GAC TG |
|  | CoatProtein F | ACT GGA GAG AGA GAT AGA GAT G |
|  | CoatProtein R | TGG CAC GAG TGT TTG AGA TGT C |
|  | HcPro ApaI F | GGA GGG CCC ATG GCC AAT GAC GTG GCT GAA AAA TTC TG |
|  | HcPro XhoI R | CCG CTC GAG TCA GCC GAC AAT GTA GTG CTT CAT T |
|  | HcPro EcoRI F | CCG GAA TTC AAT GAC GTG GCT GAA AAA TTC TG |
|  | HcPro PstI R | CCG CTG CAG TCA GCC GAC AAT GTA GTG CTT CAT T |
|  | 3’ PRSV F | GTG TYT GGG THA TGA TGG A |
|  | 3’PRSV R | GTT GCG CAT ACT CAG AG |
|  | GFP F | GAT GGC CCT GTC CTT TTA CC |
|  | GFP R | CTC TCT TTT CGT TGG GAT CTT TC |
