# Supplementary material for: Inhibition of the Host Proteasome Facilitates Papaya Ringspot Virus Accumulation and Proteosomal Catalytic Activity Is Modulated by Viral Factor HcPro
Source: PLoS One. 2012 Dec 27;7(12):e52546. doi: 10.1371/journal.pone.0052546 (PMC3531422; doi:10.1371/journal.pone.0052546)
Supplement: Table S2 — In silico analysis of PRSV protein sequences to evaluate the stability of the protein based on the N-end rule and presence of PEST sequence. (DOC) [file pone.0052546.s003.doc]

| **Name of the protein** | **Total amino acid** | **Molecular weight (kDa)** | **Starting amino acid** | **Instability index** | **Stability** | **Potential PEST sequence**  **(position)** | **N end rule** |
| --- | --- | --- | --- | --- | --- | --- | --- |
| PI | 546 | 62.6 | M | 45.25 | unstable | Yes  (184 -196 aa) | Stabilising |
| HcPro | 457 | 51.9 | N | 35.26 | stable | no | Tertiary de-stabilising residue |
| P3 | 344 | 39.8 | G | 39.47 | stable | no | Stabilising |
| 6KI | 53 | 6.1 | Q | 14.38 | stable | no | Tertiary de-stabilising residue |
| CI | 635 | 71.3 | S | 37.04 | stable | no | Stabilising |
| 6K2 | 57 | 6.4 | G | 5.59 | stable | no | Stabilising |
| VPg | 189 | 21.4 | G | 33.59 | stable | no | Stabilising |
| NIa-Pro | 238 | 26.4 | G | 25.71 | stable | no | Stabilising |
| NIb | 536 | 61.7 | S | 39.36 | stable | no | Stabilising |
| CP | 286 | 32.8 | Q | 35.30 | stable | no | Tertiary de-stabilising residue |
